# Do clinical decision-support reminders for medical providers improve isoniazid preventative therapy prescription rates among HIV-positive adults?: study protocol for a randomized controlled trial

**DOI:** 10.1186/s13063-015-0558-8

**Published:** 2015-04-09

**Authors:** Eric P Green, Caricia Catalani, Lameck Diero, E Jane Carter, Adrian Gardner, Charity Ndwiga, Aggrey Keny, Philip Owiti, Dennis Israelski, Paul Biondich

**Affiliations:** Kijani Consulting, 1289 Fordham Blvd, Suite 165, Chapel Hill, 27514 NC USA; InSTEDD, 955 Benecia Ave, Sunnyvale, 94085 CA USA; Academic Model Providing Access to Healthcare (AMPATH), Eldoret, PO Box 460630100 Kenya; Division of Medicine, Alpert Medical School of Brown University, Box G-A, Providence, 02912 RI USA; Population Council, General Accident House, Ralph Bunche Road, Nairobi, Kenya; Regenstrief Institute, 1050 Wishard Blvd, Indianapolis, 46202 IN USA; Duke Global Health Institute, Durham, PO Box 9051927708 NC USA; School of Medicine, Stanford University, 291 Campus Dr, Stanford, 94305 CA USA; Department of Medicine, School of Medicine, College of Health Sciences, Moi University, Eldoret, PO Box 4606 030100 Kenya; Department of Medicine, School of Medicine, Indiana University, 340 W 10th St 6200, Indianapolis, 46202 IN USA; School of Public Health, University of California, Berkeley, University Hall, Berkeley, 94720-7360 CA USA

**Keywords:** Tuberculosis, HIV, Isoniazid, Kenya, eHealth, Electronic medical record, Clinical decision support

## Abstract

**Background:**

This document describes a research protocol for a study designed to estimate the impact of implementing a reminder system for medical providers on the use of isoniazid preventative therapy (IPT) for adults living with HIV in western Kenya. People living with HIV have a 5% to 10% annual risk of developing active tuberculosis (TB) once infected with TB bacilli, compared to a 5% lifetime risk in HIV-negative people with latent TB infection. Moreover, people living with HIV have a 20-fold higher risk of dying from TB. A growing body of literature suggests that IPT reduces overall TB incidence and is therefore of considerable benefit to patients and the larger community. However, in 2009, of the estimated 33 million people living with HIV, only 1.7 million (5%) were screened for TB, and about 85,000 (0.2%) were offered IPT.

**Methods/Design:**

This study will examine the use of clinical decision-support reminders to improve rates of initiation of preventative treatment in a TB/HIV co-morbid population living in a TB endemic area. This will be a pragmatic, parallel-group, cluster-randomized superiority trial with a 1:1 allocation to treatment ratio. For the trial, 20 public medical facilities that use clinical summary sheets generated from an electronic medical records system will participate as clusters. All HIV-positive adult patients who complete an initial encounter at a study cluster and at least one return encounter during the study period will be included in the study cohort. The primary endpoint will be IPT prescription at 3 months post the initial encounter. We will conduct both individual-level and cluster-level analyses. Due to the nature of the intervention, the trial will not be blinded. This study will contribute to the growing evidence base for the use of electronic health interventions in low-resource settings to promote high-quality clinical care, health system optimization and positive patient outcomes.

**Trial registration** ClinicalTrials.gov NCT01934309, registered 29 August 2013.

## Background

People living with HIV have a 5% to 10% annual risk of developing tuberculosis (TB) disease once infected with TB bacilli, compared to a 5% lifetime risk in HIV-negative people with latent TB infection. Moreover, people living with HIV have a 20-fold higher risk of dying from TB [1]. Active TB disease occurs at any stage of HIV infection and is often the first recognized presentation of the underlying viral infection [2,3]. As such, routinely screening for TB during HIV care creates important opportunities to diagnose and promptly treat active disease, and also to identify those without active TB disease who are eligible for isoniazid preventative therapy (IPT) [4]. A growing body of literature suggests that IPT reduces overall TB incidence and is therefore of considerable benefit to patients and the larger community [5]. For these reasons, the World Health Organization recommends routine, repeated clinical screening for active TB disease among all people living with HIV and the provision of either treatment for active disease or IPT for asymptomatic patients to mitigate the risk of developing active TB [6].

However, in 2009, of the estimated 33 million people living with HIV, only 1.7 million (5%) were screened for TB, and about 85,000 (0.2%) were offered IPT [7]. Greatly improving TB screening, diagnosis and treatment for people living with HIV will require deployment of an effective means of triaging and monitoring this group of patients. While the use of alerts and reminders and associated health informatics solutions to enhance health service delivery is generally well described, this study, to our knowledge, is the first to look at the use of clinical decision-support reminders to directly improve rates of TB screening and initiation of preventative treatment in a TB/HIV co-morbid population.

### Current study

Indiana University, Moi University School of Medicine, the Moi Teaching and Referral Hospital and several other medical school partners have created in western Kenya an innovative and comprehensive response to HIV/AIDS and other health challenges through their support of the Kenyan Ministry of Health. This consortium, the Academic Model Providing Access to Healthcare (AMPATH), promotes and fosters a comprehensive approach to HIV/AIDS control that complements and enhances the existing Kenyan Ministry of Health infrastructure. This initiative supported by the United States Agency for International Development (USAID) strengthens health systems. It addresses food and income security needs, delivers and monitors antiretroviral treatment, and fosters prevention of HIV transmission through community-based health education and prevention of maternal-to-child transmission. Importantly, AMPATH works with all levels of health providers, from the highest levels of government to community health volunteers (CHVs), to provide effective and culturally appropriate care.

The HIV burden in AMPATH’s catchment is large (greater than 15% in some areas). There is a corresponding large clinical enterprise in support of HIV care, which collectively manages approximately 40,000 HIV patient visits per month. Additionally, TB risks to this population are substantial. Nationally, prevalence and incidence rates of TB were estimated to be 299 and 272, respectively, per 100,000 in 2012 [8]. Within the AMPATH network, approximately 45% of patients with active TB have HIV co-infection.

#### Objective

The overall research objective is to evaluate the impact of implementing a reminder system for medical providers (i.e., nurses, clinical officers, medical officers and consultants) to improve TB case-finding and the use of IPT for adults living with HIV in western Kenya. Our main research question is as follows:

Does providing medical personnel with patient-specific reminders regarding TB that are generated from a patient’s electronic medical record and based on clinical algorithms for screening significantly increase IPT prescription rates and decrease the time from initial encounter to IPT initiation?

This study is designed to contribute to the growing evidence base for the use of electronic health (eHealth) interventions to promote high-quality clinical care, health system optimization, and positive patient outcomes. Because this intervention is based on an open-source electronic medical record (EMR) system created specifically for low-resource settings, it is reasonable to conclude that positive results could be replicated in other locations that invest in the basic infrastructure needed to support this system.

## Methods/Design

### Intervention

The intervention to be studied involves providing clinic-based medical care providers (e.g., physicians, nurses and clinical support staff) with patient-specific clinical reminders regarding TB that are generated from a patient’s EMR and based on accepted clinical algorithms for TB screening and treatment.

#### Electronic medical records

The back end of AMPATH’s existing clinical decision-support system (CDSS) is an EMR system that is capable of tracking millions of patient encounters. The AMPATH Medical Record System (AMRS) is one of the world’s largest implementations of the Open Medical Records System (OpenMRS) [9]. AMRS includes more than 100 million clinical observations from more than 300,000 patients. The system tracks data on test results, patient history, physical exam findings, treatment plans, adherence and related information from clinical encounters.

AMRS is optimized for rural western Kenya. Medical personnel complete paper encounter forms at every patient visit, and centralized teams of data clerks enter the data into AMRS [10]. Pilot studies are underway to guide the transition to electronic data entry at the point of care, but currently records are digitized centrally. This makes it feasible to extend the coverage of the EMR system to include the most remote facilities that do not have reliable power or access to computers or tablets.

#### Clinical decision support

Prior to a patient’s next scheduled visit, clinic personnel print a paper clinical summary sheet that gives vital details and patient-specific care suggestions, which are automatically generated by the system based on a defined set of rules [11]. This summary sheet promotes effective task shifting as it helps providers with modest levels of training to provide high-quality standardized care [12].

There are currently more than 60 possible clinical care reminders that could be triggered and printed on a patient’s summary sheet, but there is only enough space to display a few of the most relevant reminders for each patient. The only TB-related reminder currently used in the system is a reminder about the need to conduct and report the results of a chest radiograph (CXR), which is usually performed at the initial encounter to rule out active pulmonary infection.

#### Tuberculosis clinical care reminders

As part of this study, we created a new set of reminders for TB prevention and treatment based on accepted clinical algorithms [13]. These reminders are designed to prompt medical providers to complete TB screening, consider initiating anti-TB treatment or IPT, and monitor adherence to treatment based on clinical data that exist in AMRS.

For example, if a patient does not have a history of TB or IPT, the results of a physical examination and patient-reported symptoms are not suggestive of active TB, and CXR results are normal, the paper summary sheet printed for the patient’s next visit will include the following reminder:

Test results do NOT suggest active TB. If patient still does NOT report TB symptoms, consider initiating IPT. IPT is effective and could save [his/her] life.

### Study design

#### Type of study

This will be a pragmatic, parallel-group, cluster-randomized superiority trial involving 20 public medical facilities in the AMPATH network in western Kenya.

#### Treatment group specification and assignment

Clusters will be sorted into four strata and randomly assigned to the treatment or control group with an allocation ratio of 1:1. The two stratifying variables will be average monthly patient volume and the percentage of initial patient encounters in 2013 with CXR results recorded in AMRS. Stratifying on cluster size is advantageous when cluster sizes vary in the population and when cluster size might be correlated with the endpoint of interest [14]. Stratifying on the availability of CXR results in AMRS will potentially reduce the imbalance on a cluster-level covariate that we expect will be related to isoniazid (INH) prescribing behavior.

AMPATH clinical protocols advise that all patients should have a normal CXR that is no more than 6 months old before medical providers prescribe IPT (assuming no other contraindications). However, in 2013, fewer than a third of initial encounters with HIV-positive adults had CXR results recorded in AMRS. When results are missing from AMRS, CDSS will print a general reminder about ordering a CXR. At best, a general reminder that prints for most patients will potentially lead to reminder fatigue. At worst (when CXR results exist and are known to the provider), the prompt to order a CXR is a false positive; in these cases, the summary sheet should contain a more specific reminder about considering anti-TB treatment or IPT based on the CXR result and patient symptoms, not a reminder to order a CXR. We expect the general reminder to order a CXR, particularly where the printing of this reminder is a false positive, to be less impactful on INH prescribing behavior. We also expect these messages to be more common at facilities that are less successful in entering CXR results into AMRS. Therefore, we will stratify randomization on baseline facility-level availability of CXR results in AMRS.

Both stratifying variables will be dichotomized (volume greater than 1,000 patients per month and percentage of results in AMRS greater than 23%) to create four possible strata: low volume/low results; low volume/high results; high volume/low results; high volume/high results.

Clinical summary sheets for patients receiving care at treatment clusters will include the new TB care reminders (as needed) *plus* the full set of existing reminders, whereas summary sheets printed at control clusters will only include the existing set of reminders (i.e., no new TB care reminders).

### Procedures

Following the current practice within AMPATH, clinical encounter data will be recorded on standard paper encounter forms by medical providers at the point of care, and the existing team of data entry clerks will enter the encounter data into AMRS. Existing data quality personnel will continue to review the data for completeness and consistency and will return problematic forms to cluster administrators for correction. Paper summary sheets with clinical decision-support reminders will be printed for each patient as usual and delivered to facilities without on-demand printing capabilities. In a departure from current practice, additional data clerks will collect data on facility-level treatment compliance, INH stock at AMPATH pharmacies, and IPT eligibility for indeterminate cases in the time window for the 3-month post-initial encounter endpoint.

### Study population

#### Medical facilities

Medical providers with responsibility for diagnosing TB and prescribing TB medication and IPT will be the intervention targets for this study. Due to logistical challenges of provider randomization and concerns about spillover between medical personnel exposed to TB reminders and personnel in the no TB reminders control group, medical facilities rather than individual providers will be randomized to the treatment and control groups.

To be included in the study as a cluster, a facility had to meet the following criteria:

- active operations as an HIV Care Clinic
- uses AMPATH HIV initial and return encounter forms (paper)
- enters data into AMRS or sends forms to central location for data entry
- prints or receives printed paper clinical summary sheets with reminders
- prescribes IPT
- classified as a mother facility in the AMPATH network^a^

There are currently 22 AMPATH facilities that meet these criteria. We decided to exclude two facilities to bring the total number of study clusters to 20: a facility that operates as a regional referral hospital and a small facility that is very new to the network.

#### Patients

To be included in the impact analysis, patients must meet the following criteria:

- HIV positive
- completed an adult initial encounter at a study cluster during the enrollment period^b^
- no history of TB or IPT
- attended at least one additional appointment within 90 days after the initial encounter at any study facility

Patients will not be recruited into the study, per se, as the intervention will be provided to medical personnel and allocated by cluster. Instead, we will run pre-specified data queries in AMRS to identify the cohort of eligible patients.

To be eligible for IPT, and thus eligible for our analysis, patients must have no evidence of active TB. Since HIV-positive adult patients in the AMPATH network typically attend monthly medical appointments, it is reasonable to assume that patients should be classified as eligible for IPT or not eligible for IPT within 2 months of their initial encounter dates. Within 3 months of the initial encounter, patients with no evidence of active TB should have been prescribed INH. Therefore, 3 months after each initial encounter, patients will be classified according to their IPT eligibility and INH prescription status at that time.

Patients who are not eligible for IPT will be excluded from the analysis dataset, while IPT-eligible patients will be included. Importantly, a cluster’s failure to classify a patient as eligible for IPT or not eligible for IPT by the 3-month mark will not exclude the patient from the analysis dataset as improving the screening and diagnostic process is a target of the intervention. Instead, any indeterminate cases at the 3-month post-initial encounter mark will be flagged by our data queries for investigation. We anticipate that the most common reason for indeterminate cases will be missing CXR results. To determine IPT eligibility for study purposes, data clerks will investigate all indeterminate cases and ask medical providers at the cluster to classify the case as eligible for IPT, not eligible for IPT, or still indeterminate. Cases ultimately determined to be eligible for IPT following this review will be included in the analysis dataset; however, these cases will be marked as failures on the primary INH prescription endpoint since, without a determination of eligibility for IPT at the 3-month mark, the patient could not initiate IPT at that time^c^.

The final eligibility criterion is that a patient must attend at least one additional appointment within 90 days of her initial encounter so that her provider has the opportunity to be exposed to the CDSS reminders on the summary sheet. Since a patient’s initial encounter is her first contact with AMPATH at the facility level, there is no summary sheet available during a patient’s initial encounter.

### Ethical considerations

This protocol has been reviewed and approved by the Moi University School of Medicine/Moi Teaching and Referral Hospital Institutional Research and Ethics Committee (0001055), the Indiana University Institutional Review Board (1307011750), and the Lifespan Research Protection Office (0000396, 00004624). The approving ethics committees did not require individual patient consent given the nature of the intervention.

### Statistical analysis

#### Study objectives as statistical hypotheses

##### Primary hypothesis

*H*_0_ There will be no measurable difference in the rate of INH prescriptions among IPT-eligible patients (i.e., patients with no evidence of active TB) who receive care from treatment or control clusters.

*H*_*a*_ The INH prescription rate among IPT-eligible patients receiving care from medical providers at treatment clusters will be greater than the INH prescription rate among IPT-eligible patients receiving care from medical providers at control clusters.

##### Secondary hypothesis

*H*_0_ There will be no measurable difference in the time lapse from initial encounter to INH prescription among IPT-eligible patients (i.e., patients with no evidence of active TB) who receive care from treatment or control clusters.

*H*_*a*_ The time lapse from initial encounter to INH prescription among IPT-eligible patients receiving care from medical providers at treatment clusters will be shorter than the time lapse from initial encounter to INH prescription among IPT-eligible patients receiving care from medical providers at control clusters.

#### Endpoints

INH will be considered prescribed when there is an entry in AMRS indicating that a medical provider prescribed INH ^d^. The denominator for the calculation of the cluster-level prescription rate will be the number of IPT-eligible patients in the study who completed an initial and return encounter during the study period. The time lapse from the date of a patient’s initial encounter to the date of INH prescription will be calculated in days. The endpoint for the INH prescription time lapse will be calculated by averaging cluster-level INH prescription lapse means among IPT-eligible patients across the treatment and control groups separately.

#### Statistical methods

There are two main approaches to the analysis of cluster-randomized trials: cluster-level analysis or regression analysis of individual-level observations that take into account intracluster correlation [15]. Individual-level regression methods are more statistically efficient and allow for the presentation of the effects of modeled covariates alongside intervention effects, but this approach may not be robust when there are a relatively small number of clusters per arm. Therefore, we will conduct both individual-level and cluster-level analyses.

##### Cluster-level summaries

To determine if there is a statistically significant difference in the proportion of INH prescriptions among IPT-eligible patients between treatment and control clusters, we will use an unpaired *t*-test. We will also estimate the relative risk of INH prescription and associated 95% confidence intervals. We will use Kaplan–Meier methods to calculate the proportion of eligible patients receiving INH prescriptions within 3 months of their initial encounters. To test the null hypothesis that there is no difference between the survival curves, we will use the log rank test.

##### Individual-level regression analysis

In addition to the analyses based on cluster-level summaries, we will also use regression models with individual-level data that account for between-cluster variation. Specifically, we will evaluate the reliability of logistic regression random effects models and generalized estimating-equation approaches to model our binary outcome. We will use Cox regression with random effects to model the time to INH prescription. Individual-level covariates will be limited to data collected reliably in AMRS, such as age, gender and CD4 count.

#### Treatment compliance

All analyses will be on an intention-to-treat basis; thus, we will analyze all observations based on the initial assignment to the study arms, not compliance with the treatment protocol. Yet, treatment compliance will be an important construct to measure in the event of null results so that we can distinguish between intervention and theory failure.

##### Cluster-level compliance

In this study, clusters (mother sites and their satellite sites) will be randomly assigned to receive the new TB reminders plus the existing CDSS reminders (treatment) or just the existing CDSS reminders (control). So to be compliant, clusters need to print correct summary sheets, deliver the sheets to all cluster facilities if printing is not done on-site, and ensure that the sheets are inserted into patients’ medical files before encounters.

A correct summary sheet is one in which TB reminders are either present or absent depending on the cluster treatment status. All reminders have been tested against hundreds of patient records to ensure accuracy. Once the trial begins, we will log all reminders printed on every summary sheet.

The next step of the compliance chain is for facilities to print the paper summary sheet prior to a patient’s next encounter. Some facilities print on-site; others have sheets delivered from the closest printing facility. Some of the printing facilities print summary sheets on-demand as patients arrive; others print in advance. Facilities that have summary sheets delivered and those that print in advance also place the papers in patient folders prior to the encounter. For reminders to be effective, the summary sheets containing the reminders must be inserted into a patient’s file prior to the encounter with the medical provider. There is no system in place currently to record that this happens for every patient encounter. We do, however, track facility-level compliance through random spot checks.

##### Medical provider behavior

Medical providers could also be a source of cluster non-compliance. For instance, a medical provider who crosses between treatment and control clusters would be non-compliant with her assigned treatment status. Since nearby sites (mother and satellite) are grouped into clusters that are then assigned to the study arms as a cluster, we are not very concerned about this possibility. Tracking where medical personnel provide care will be possible through queries of AMRS; the location of all encounters is recorded on every encounter form.

Another source of medical provider non-compliance would occur when a provider does not read the summary sheet that is placed in a patient’s file. Determining whether or not a provider has read a summary sheet and the reminders is a challenge. At best, we could have providers check a box indicating that they reviewed a summary sheet, but (a) this does not mean that a review actually occurred and (b) we will not know what a missing check indicates (i.e., a lack of a review or a review with a missing check).

It is important to note that treatment compliance is not whether providers act upon printed reminders. Rather, provider behavior (screening for IPT eligibility and prescribing INH when indicated) is part of our study endpoint.

##### Patient-level compliance

Reminders will be administered at the cluster level, but our primary outcome (INH prescription) will be observed at the patient level. Like medical providers, patients can be non-compliant if they cross between treatment and control clusters to receive care. However, an analysis of 2012 AMPATH data suggests that encounters at multiple sites are rare. When multiple-site treatment does occur, it is usually within a single cluster of mother and satellite sites, thus avoiding the risk of contamination. Nevertheless, encounter locations will be tracked.

#### Subset analyses

Previous research in the AMPATH network suggests that IPT initiators are more likely to be younger, female and have a higher CD4 count. We will dichotomize each variable and test for differing intervention effects in each subgroup. We will also test the hypothesis that patients with a greater number of reminders printed on each summary sheet will be less likely to initiate IPT compared to patients with fewer reminders. It is possible that each additional reminder increases the cognitive demand on a provider and makes it more likely that the provider will ignore the reminders.

#### Statistical design considerations

This will be a pragmatic, parallel-group, cluster-randomized superiority trial with a 1:1 allocation to treatment ratio from within four strata. Assuming a desired power of 0.80, alpha of 0.05, 20 clusters equally allocated to treatment and control, 50 patients per cluster, a between-cluster coefficient of variation of 0.25 and a baseline INH prescription rate of 15%, we will be able to detect a minimum shift of almost 8 percentage points in the INH prescription rate, from 15% to almost 23% using a one-tailed test^e^.

Based on a recent retrospective review of AMRS data, we believe that the current rate of INH prescription is between 5% and 25%. Historically, this rate was higher at nearly 40%. No matter where the true baseline rate is in this range, our minimum detectable effect is not projected to rise above 8 percentage points, if everything else is held constant.

The between-cluster coefficient of variation is also important in this calculation. Without cluster-level baseline data on the INH prescription rate, we have to estimate. This variation is often less than 0.25 and rarely exceeds 0.50 [15]. In the previous calculations, we set the between-cluster variation to be 0.25 and scaled it down by 0.75 to account for the benefits of stratification. If the between-cluster variation is really at the high end of this range before scaling (0.50), then the minimum detectable effect climbs to almost 12 percentage points. If we further assume that it is only possible to observe half as many patients per cluster (25), the minimum detectable effect increases to 14 percentage points.

## Discussion

One threat to this research design is control group contamination: if patients who have an initial encounter with a control cluster later receive care from a treatment cluster, this would attenuate the treatment contrast and make it less likely that we will detect a significant treatment effect. Another threat is spillover effects that could occur if medical providers stationed at treatment clusters move to control clusters and influence clinical care at these locations. It is not clear what the effect of this scenario would be in practice. It is plausible that visits by treatment group providers could make control group providers more aware of TB screening and treatment, thus attenuating the treatment effect. It is also plausible that visiting treatment group providers could misinterpret the meaning of the lack of TB reminders at the control location and assume that no reminders indicates no need for TB-related care, thus delaying or denying IPT initiation and artificially increasing the treatment effect.

To limit both possibilities, we will restrict eligibility to clusters with a low potential for contamination and spillover due to geography. This should be sufficient because these threats, while serious, are thought to be low risk to begin with. For instance, an analysis of 2012 patient encounters suggests that fewer than 9% of patients received care at different facilities, and these instances of movement were most likely between mother and satellite sites within the same clusters. Incidents of contamination and potential spillover will be tracked and summarized.

A third threat to the research design is the loss of study clusters. Given the relatively small number of planned clusters, any reduction in study clusters after the start of the trial will reduce our power to detect the effects of the intervention. This is unlikely to be an issue.

## Trial status

The reminders intervention launched on 11 April 2014 and remains active.

## Endnotes

^a^ Mother facilities tend to be open more days each week, offer more advanced services and experience higher patient volumes than the smaller satellite facilities. Satellite facilities tend to be staffed by the same providers that operate the mother facilities and are located in close geographical proximity. It is common for patients and providers to move between a mother facility and its associated satellite facilities.

^b^ Patient volume over time will determine whether we also include patients who complete an initial encounter at a cluster’s satellite facilities.

^c^ We will conduct sensitivity analyses to determine if the results change based on whether or not we also include still indeterminate cases in the denominator of the INH prescription rate calculation.

^d^ We will also attempt to collect data on IPT initiation as measured by a patient picking up her initial batch of INH from a pharmacy. Pharmacy records are not linked to AMRS, however, and it is not clear that we can collect complete and accurate data on initiation. Prescription is a better endpoint for this study, however, as the reminders intervention targets medical providers not patients, and prescribing INH is a provider behavior.

^e^ Using Equation 7.14 for stratified cluster randomized trials [15].

## Abbreviations

AIDS: acquired immune deficiency syndrome
AMPATH: Academic Model Providing Access to Healthcare
AMRS AMPATH: Medical Record System
CDSS: clinical decision-support system
CHV: community health volunteer
CXR, chest radiograph; eHealth: electronic health
EMR: electronic medical record
HIV: human immunodeficiency virus
INH: isoniazid
IPT: isoniazid preventative therapy
OpenMRS: Open Medical Records System
TB: tuberculosis
USAID: United States Agency for International Development
